# Unjustifiable Use of Chloroform

**Published:** 1883-10

**Authors:** 


### Unjustifiable Use of Chloroform.

A death under chloroform is announced to have taken place in England, the patient having been anesthetized for the purpose of removing a tumor from the lip. Considering the ease with which local anesthesia can be produced by ether spray, and the perfect safety of this method, we are inclined to the opinion that a surgeon who administers chloroform by inhalation in cases admitting of local anesthesia should be held under a criminal charge when the result is fatal.

